# Autophagic Cell Death and Cancer

**DOI:** 10.3390/ijms15023145

**Published:** 2014-02-21

**Authors:** Shigeomi Shimizu, Tatsushi Yoshida, Masatsune Tsujioka, Satoko Arakawa

**Affiliations:** Department of Pathological Cell Biology, Medical Research Institute, Tokyo Medical and Dental University, 1-5-45 Yushima, Bunkyo-ku, Tokyo 113-8510, Japan; E-Mails: yoshida.pcb@mri.tmd.ac.jp (T.Y.); tsujioka.pcb@mri.tmd.ac.jp (M.T.); arako.pcb@mri.tmd.ac.jp (S.A.)

**Keywords:** programmed cell death, autophagic cell death, Atg5-independent autophagy, Beclin1, tumorigenesis, c-Jun *N*-terminal kinase (JNK)

## Abstract

Programmed cell death (PCD) is a crucial process required for the normal development and physiology of metazoans. The three major mechanisms that induce PCD are called type I (apoptosis), type II (autophagic cell death), and type III (necrotic cell death). Dysfunctional PCD leads to diseases such as cancer and neurodegeneration. Although apoptosis is the most common form of PCD, recent studies have provided evidence that there are other forms of cell death. One of such cell death is autophagic cell death, which occurs via the activation of autophagy. The present review summarizes recent knowledge about autophagic cell death and discusses the relationship with tumorigenesis.

## 1. Introduction

Apoptosis is a form of programmed cell death (PCD), and its molecular basis is well understood. However, PCD is mediated by other mechanisms as well. For example, morphological analyses of embryogenesis revealed three types of PCD as follows: apoptosis (type I cell death), autophagic programmed cell death (type II cell death), and necrotic programmed cell death (type III cell death) [1,2]. This review focuses on recent experimental evidence that illuminates the mechanisms of autophagic cell death and its role in tumorigenesis.

## 2. Apoptosis

**Figure 1.**
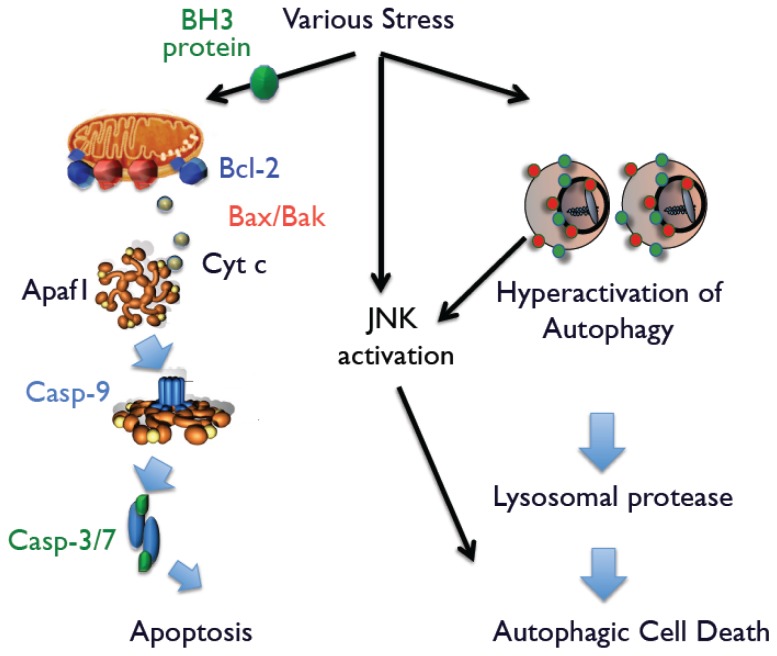
Molecular mechanism of apoptosis and autophagic cell death. An increase in the permeability of the outer mitochondrial membrane is crucial for apoptosis to occur and is regulated by multidomain pro-apoptotic members of the Bcl-2 family (Bax and Bak), resulting in the release of cytochrome *c* into the cytoplasm. Then, cytochrome *c* associates with apoptotic protease activating factor-1 (Apaf-1), which activates the caspase cascade to execute apoptotic cell death. Apoptosis-associated mitochondrial membrane permeability is primarily controlled by Bcl-2 family members. When apoptosis is blocked, various apoptotic stimuli activate autophagy and c-Jun *N*-terminal kinase (JNK), resulting in the induction of autophagic cell death.

Apoptosis is the most crucial form of cell death in mammals, and its molecular signaling pathway is well defined [3]. Mammalian cells possess two apoptotic signaling pathways, which are known as the intrinsic and extrinsic pathways. In the intrinsic pathway, most apoptotic stimuli transmit death signals to the mitochondria and increase the permeability of the outer mitochondrial membrane, which leads to the release of apoptogenic proteins, such as cytochrome *c*, Smac, and Omi, into the cytoplasm from the mitochondria. After its release, cytochrome *c* associates with apoptotic protease activating factor-1 (Apaf-1), which activates the caspase cascade to execute apoptotic cell death. Smac and Omi accelerate caspase activation through inhibition of Inhibition of Apoptosis (IAP) family members that function as endogenous caspase inhibitors. Apoptosis-associated mitochondrial membrane permeability is primarily controlled by Bcl-2 family members, which include three subgroups: anti-apoptotic proteins (Bcl-2, Bcl-x_L_, and Mcl-1); multi-domain pro-apoptotic proteins (Bak and Bax); and proteins with only BH3 domain (Bid, Bim, Bik, Bad, Noxa, and Puma). Among them, Bak and Bax play an essential role in the release of apoptogenic proteins (Figure 1). In the extrinsic pathway, engagement of death receptors, such as Fas and tumor necrosis factor-α receptor, directly activates caspase-8 that activates downstream caspases, such as caspase-3 or caspase-7, which induce apoptotic cell death.

Until 10 years ago, evidence indicated that physiological and pathological cell death was primarily mediated through apoptosis. However, detailed analysis of Bax/Bak double-knockout mice in which the intrinsic apoptotic pathway is totally inhibited, indicated that PCD largely proceeds normally, even in mice resistant to apoptosis [4,5]. These findings focused the attention of researchers on identifying other pathways of PCD, which led to the discovery that autophagy also mediates PCD as well as other physiological and pathological events.

## 3. Autophagy

Autophagy is a catabolic process that digests cellular contents within lysosomes. Autophagy is a low-level constitutive function that is accelerated by a variety of cellular stressors such as nutrient starvation, DNA damage, and organelle damage. Autophagy serves as a protective mechanism that facilitates the degradation of superfluous or damaged cellular constituents, although hyperactivation of autophagy can lead to cell death.

Evidence supports the conclusion that autophagy represents the major pathway for degrading cytoplasmic proteins and organelles [6]. In this multistep process, cytoplasm and damaged organelles are sequestered inside isolation membranes that eventually mature into double-membrane structures called autophagosomes that subsequently fuse with lysosomes to form autolysosomes, which digest the sequestered components. The molecular basis of autophagy was first studied in autophagy-defective mutant yeasts [6]. Subsequent identification of vertebrate homologs of yeast autophagy proteins greatly expanded our understanding of the molecular mechanisms of autophagy.

**Figure 2.**
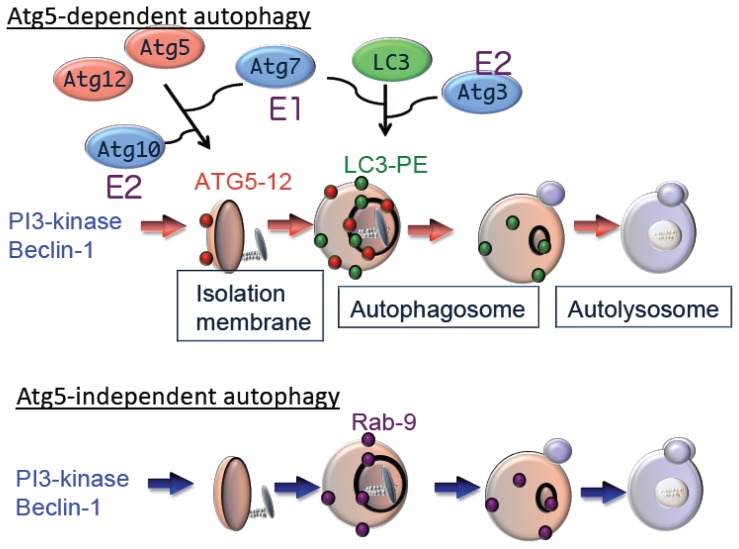
Hypothetical model of macroautophagy. There are at least two modes of macroautophagy, *i.e.*, conventional and alternative macroautophagy. Conventional macroautophagy depends on Atg5 and Atg7, is associated with light chain 3 (LC3) modification and may originate from Endoplasmic reticulum (ER)-mitochondria contact membrane. In contrast, alternative macroautophagy occurs independent of Atg5 or Atg7 expression and LC3 modification. The generation of autophagic vacuoles in this type of macroautophagy may originate from Golgi membrane and late endosomes (LE) in a Rab9-dependent manner. Although both these processes lead to bulk degradation of damaged proteins or organelles by generating autolysosomes, they seem to be activated by different stimuli, in different cell types and have different physiological roles.

Autophagy is driven by more than 30 well-conserved proteins (Atgs), from yeasts to mammals [7]. Atg1 (also called Unc51-like kinase 1 (Ulk1)) is a serine/threonine kinase essential for the initiation of autophagy [8]. Autophagy is also regulated by phosphatidylinositol 3-kinase (PI3K) type III, which is a component of a multi-protein complex that includes Atg6 (Beclin1). PI3K promotes invagination of the membrane at domains rich in phosphatidylinositol-3-phosphate (PI3P), which are called omegasomes, to initiate generation of the isolation membrane [9]. Subsequent expansion and closure of isolation membranes are mediated by two ubiquitin-like conjugation pathways as follows: the Atg5–Atg12 pathway and the microtubule-associated protein 1 light chain 3 (LC3) pathway [7]. Ubiquitin-like conjugation of phosphatidylethanolamine (PE) to LC3 facilitates translocation of LC3 from the cytosol to sites of origin of the autophagic membrane. Moreover, the localization of LC3-PE in autophagic membranes makes this complex a reliable marker of autophagy. The suppression of autophagy in Atg5-deficient cells indicates the essential role of the Atg5–Atg12 pathway in autophagy (Figure 2).

Although Atg5 function is crucial for autophagy, we identified an Atg5-independent autophagic pathway, which is induced when cells are severely stressed, for example, by DNA damage but not by nutrient deprivation. The morphology of Atg5-independent autophagic structures is indistinguishable from those that form during Atg5-dependent autophagy, *i.e.*, isolation membrane, autophagosomes, and autolysosomes [10]. We call the Atg5-independent pathway alternative macroautophagy, which is driven by Ulk1 and phosphoinositol 3-kinase complexes at the initiation steps. These molecules function at the initiation of conventional autophagy as well. In contrast, this pathway is not mediated by other components, such as Atg9 and proteins of the ubiquitin-like protein conjugation system (Atg5, Atg7, and LC3), that function to extend conventional autophagic membranes (Figure 2). Because alternative macroautophagy requires the extension of autophagic membranes, several molecules may serve to mediate this function. One such molecule is Rab9, a protein involved in membrane trafficking. Experiments using dominant negative mutants and gene silencing found that Rab9 is essential for membrane expansion and fusion in alternative macroautophagy.

Alternative macroautophagy is not an atypical form of autophagy because it occurs in a wide variety of cells, including embryonic fibroblasts and thymocytes as well as in various tissues such as that of the heart, brain, and liver. It is activated by a variety of stressors but not by rapamycin. Erythrocyte maturation is a representative example of physiological events that involve alternative macroautophagy. Enucleation and the clearance of mitochondria occur during the transition of erythroblasts into reticulocytes, which occurs prior to terminal maturation into erythrocytes, with macroautophagy functioning in the latter process. In contrast, erythrocyte maturation usually occurs in Atg5-deficient embryos [10], indicating that conventional macroautophagy does not eliminate mitochondria from erythrocytes.

Moreover, ultrastructural analysis has demonstrated that autophagic structures in reticulocytes equivalently engulf and digest mitochondria in wild-type and Atg5-deficient embryos. Furthermore, mitochondrial clearance from erythrocytes is significantly altered in Ulk1-deficient embryos in the absence of macroautophagy [11]. Therefore, these data suggest that Ulk1-mediated alternative macroautophagy plays a crucial role in the elimination of mitochondria from erythrocytes. Ongoing research promises to provide a more detailed picture of the physiological and pathological relevance of alternative macroautophagy. In addition to alternative macroautophagy, previous reports have described a Beclin 1-independent type of autophagy [12]. Although conventional, alternative, and Beclin 1-independent macroautophagy generally leads to bulk degradation of cellular proteins, they may also be activated by various stimuli in different cell types and play different physiological roles. For a better understanding of macroautophagy, it is important to classify autophagy-related molecules according to the type of macroautophagy in which they are involved.

## 4. Autophagic Cell Death

Although many autophagic cells are present in regions where cell death occurs, certain modes of cell death that are accompanied by protective autophagy are not called “autophagic cell death”. For example, a large number of autophagic cells are observed when cells are starved. However, autophagy prevents starvation-induced necrosis and does not induce cell death because the inhibition of autophagy accelerates, rather than inhibiting, cell death [13]. Therefore, we suggest using the term “autophagic cell death.” based on molecular and functional aspects, to indicate cell death mediated by autophagy. The term autophagic cell death should be used when cell death is suppressed by the inhibition of autophagy using chemical inhibitors (e.g., 3-methyl adenine and wortmannin) or genetic ablation (e.g., knockout or siRNA silencing of essential autophagy genes). If inhibiting autophagy does not prevent cell death, this process should not be called autophagic cell death.

There is skepticism regarding the phenomenon of mammalian autophagic cell death because Atg5-deficient mice generally do not show any developmental defects [14]. However, as described earlier, mammalian cells are capable of performing two types of autophagy (Atg5-dependent and Atg5-independent) and their functions compensate each other. Thus, the absence of abnormalities in Atg5-deficient embryos does not necessarily indicate the absence of autophagic cell death. There are several distinct settings of autophagic cell death. First, caspase-independent, autophagy-dependent PCD occurs during mammalian embryogenesis. Second, autophagy may induce developmental cell death during regression of the salivary glands in *Drosophila* [15]. Third, autophagy-mediated cell death is induced in apoptosis-resistant cells, particularly in the absence of the pro-apoptotic proteins Bax and Bak [16,17]. The latter situation should provide an excellent experimental system to investigate autophagic cell death, because the process can be observed at the cellular level.

## 5. Molecular Mechanism of Autophagic Cell Death

Bax/Bak-deficient cells do not undergo apoptosis after exposure to a variety of apoptotic stimuli, although these cells still die with numerous autophagic structures [16]. This type of cell death is inhibited by autophagy inhibitors or by silencing autophagy genes such as Atg5 and Atg6 [16]. Thus, Atg5-dependent autophagy is required for the death of Bax/Bak-deficient cells after exposure to apoptotic stimuli (Figure 2). Although we discovered Atg5-independent alternative autophagy, it is not involved in autophagic cell death of Bax/Bak-deficient cells. However, this does not necessarily indicate that alternative autophagy in autophagic cell death is irrelevant. In other situations, alternative autophagy potentially induces autophagic cell death.

Because activation of autophagy is necessary but not sufficient for autophagic cell death, it requires additional death signals. We recently discovered that c-Jun N-terminal kinase (JNK) generates such death signals [13]. When Bax/Bak-deficient cells were exposed to apoptotic stimuli, we found that autophagic cell death occurs simultaneously with increased levels of phosphorylated JNK. The essential role of phospho-JNK was confirmed by the addition of JNK inhibitors or the enforced expression of a dominant-negative (DN) JNK plasmid. Interestingly, neither the JNK inhibitor nor JNK-DN influence the extent of autophagy, suggesting that simultaneous activation of JNK and autophagy is required for autophagic cell death. Supporting this conclusion are findings that nutrient-starved cells possess large numbers of autophagic structures. However, because JNK was not activated in these cells, cell death was not caused by autophagy. Further, enforced expression of activated JNK in these cells rapidly induced autophagy-mediated cell death. Thus, we suggest that activation of autophagy and JNK is necessary when cells are committed to autophagic cell death (Figure 1).

## 6. Cancer and Autophagic Cell Death

It has been suggested that autophagic cell death participates in physiological and pathological events. Although a large body of evidence indicates that the inhibition of apoptosis is critical for tumorigenesis, elimination of cancer cells may also be mediated through autophagic cell death and there is evidence that decreased autophagic activity is related to tumorigenesis. For example, Beclin1 (Atg6) levels are lower in ovarian, breast, and prostate cancer because of monoallelic mutations [18]. Furthermore, mice heterozygous for the beclin1 gene have a higher risk for tumorigenesis [18,19]. Therefore, these findings provide compelling evidence that inhibition of Beclin1 expression contributes to the pathogenesis of cancer. Moreover, previous reports have shown that loss-of-function mutations of *Atg5* and *LC3* promote myeloma and glioblastoma, respectively [20,21], suggesting that failure of cells to undergo autophagy leads to tumor progression. Although autophagy plays a role in tumor suppression, it also promotes tumor cell growth by supplying nutrients under hypoxic conditions. Cancer cells require several nutrients to accelerate cell proliferation, and studies have shown that nutrient deprivation results in the impairment of tumor growth in some autophagy-deficient cancer cells [22]. Thus, these research findings suggest that autophagy plays dual roles in cancer and well as functions in a context-dependent manner.

Several mechanisms have been proposed to explain the tumor suppressive function of autophagy: (1) accumulation of p62, a substrate of autophagy, leads to NF-κB activation [22]; (2) accumulation of p62 stabilize Nrf2, which imparts tumor cells with resistance to hypoxic stress [23]; (3) retention of damaged organelles, including mitochondria, increases the level of active oxygen species and increases the mutation rate; and (4) defective elimination of cancer cells due to the loss of autophagic cell death [13]. These mechanisms may be cell- and stimulus-type specific; however, we believe that it is reasonable to conclude that the failure of autophagic cell death is one of the most crucial mechanisms for tumorigenesis because autophagic cell death occurs in normal cells (e.g., fibroblasts or thymocytes) but not in most cancer cells. Furthermore, in some cancer cells, the magnitude of JNK activation, a crucial factor for autophagic cell death, is significantly lower compared with that in normal cells after exposure to apoptotic stimuli [13]. In such cancer cells, JNK activity may not reach the threshold level required to induce autophagic cell death. This conclusion is supported by data showing that enforced expression of activated JNK in cancer cells induces autophagic cell death. Taken together, it is likely that insufficient activation of JNK followed by failure of autophagic cell death may induce uncontrolled growth of cells that ultimately acquire the malignant phenotype.

## 7. Conclusions and Future Prospects

Here we describe the molecular mechanism and pathological role of autophagic cell death; however, the underlying mechanisms remain to be defined in detail. Moreover, the lack of molecular markers of autophagic cell death impedes research progress. Therefore, defining the biological roles of autophagic cell death will require a better understanding of the relevant molecular mechanisms of autophagic cell death, and the contribution of autophagic cell death to physiology and pathogenesis, particularly tumorigenesis.

## References

[1] Clarke P.G. (1990). Developmental cell death: Morphological diversity and multiple mechanisms. Anat. Embryol. (Berl.).

[2] Baehrecke E.H. (2002). How death shapes life during development. Nat. Rev. Mol. Cell Biol.

[3] Tsujimoto Y. (2003). Cell death regulation by the Bcl-2 protein family in the mitochondria. J. Cell. Physiol.

[4] Lindsten T., Ross A.J., King A., Zong W.X., Rathmell J.C., Shiels H.A., Ulrich E., Waymire K.G., Mahar P., Frauwirth K. (2000). The combined functions of proapoptotic Bcl-2 family members bak and bax are essential for normal development of multiple tissues. Mol. Cell.

[5] Wei M.C., Zong W.X., Cheng E.H., Lindsten T., Panoutsakopoulou V., Ross A.J., Roth K.A., MacGregor G.R., Thompson C.B., Korsmeyer S.J. (2001). Proapoptotic BAX and BAK: A requisite gateway to mitochondrial dysfunction and death. Science.

[6] Nakatogawa H., Suzuki K., Kamada Y., Ohsumi Y. (2009). Dynamics and diversity in autophagy mechanisms: Lessons from yeast. Nat. Rev. Mol. Cell Biol.

[7] Mizushima N., Yoshimori T., Ohsumi Y. (2011). The role of Atg proteins in autophagosome formation. Annu. Rev. Cell Dev. Biol.

[8] Kabeya Y., Kamada Y., Baba M., Takikawa H., Sasaki M., Ohsumi Y. (2005). Atg17 functions in cooperation with Atg1 and Atg13 in yeast autophagy. Mol. Biol. Cell.

[9] Axe E.L., Walker S.A., Manifava M., Chandra P., Roderick H.L., Habermann A., Griffiths G., Ktistakis N.T. (2008). Autophagosome formation from membrane compartments enriched in phosphatidylinositol 3-phosphate and dynamically connected to the endoplasmic reticulum. J. Cell Biol.

[10] Nishida Y., Arakawa S., Fujitani K., Yamaguchi H., Mizuta T., Kanaseki T., Komatsu M., Otsu K., Tsujimoto Y., Shimizu S. (2009). Discovery of Atg5/Atg7-independent alternative macroautophagy. Nature.

[11] Kundu M., Lindsten T., Yang C.Y., Wu J., Zhao F., Zhang J., Selak M.A., Ney P.A., Thompson C.B. (2008). Ulk1 plays a critical role in the autophagic clearance of mitochondria and ribosomes during reticulocyte maturation. Blood.

[12] Chu C.T., Zhu J., Dagda R. (2007). Beclin 1-independent pathway of damage-induced mitophagy and autophagic stress: Implications for neurodegeneration and cell death. Autophagy.

[13] Shimizu S., Konishi A., Nishida Y., Mizuta T., Nishina H., Yamamoto A., Tsujimoto Y. (2010). Involvement of JNK in the regulation of autophagic cell death. Oncogene.

[14] Shen S., Kepp O., Kroemer G. (2012). The end of autophagic cell death?. Autophagy.

[15] Baehrecke E.H. (2003). Autophagic programmed cell death in Drosophila. Cell Death Differ.

[16] Shimizu S., Kanaseki T., Mizushima N., Mizuta K., Arakawa-Kobayashi S., Thompson C.B., Tsujimoto Y. (2004). A role of Bcl-2 family of proteins in nonapoptotic programmed cell death dependent on autophagy genes. Nat. Cell Biol.

[17] Yu L., Alva A., Su H., Dutt P., Freundt E., Welsh S., Baehrecke E.H., Lenardo M.J. (2004). Regulation of an ATG7-Beclin 1 program of autophagic cell death by caspase-8. Science.

[18] Qu X., Yu J., Bhagat G., Furuya N., Hibshoosh H., Troxel A., Rosen J., Eskelinen E., Mizushima N., Ohsumi Y. (2003). Promotion of tumorigenesis by heterozygous disruption of the *beclin 1* autophagy gene. J. Clin. Invest.

[19] Yue Z., Jin S., Yang C., Levine A.J., Heintz N. (2003). Beclin 1, an autophagy gene essential for early embryonic development, is a haploinsufficient tumor suppressor. Proc. Natl. Acad. Sci. USA.

[20] Iqbal J., Kucuk C., deLeeuw R.J., Srivastava G., Tam W., Geng H., Klinkebiel D., Christman J.K., Patel K., Cao K. (2009). Genomic analyses reveal global functional alterations that promote tumor growth and novel tumor suppressor genes in natural killer-cell malignancies. Leukemia.

[21] Huang X., Bai H.M., Chen L., Li B., Lu Y.C. (2010). Reduced expression of LC3B-II and Beclin 1 in glioblastoma multiforme indicates a down-regulated autophagic capacity that relates to the progression of astrocytic tumors. J. Clin. Neurosci.

[22] Mathew R., Karp C.M., Beaudoin B., Vuong N., Chen G., Chen H.Y., Bray K., Reddy A., Bhanot G., Gelinas C. (2009). Autophagy suppresses tumorigenesis through elimination of p62. Cell.

[23] Takamura A., Komatsu M., Hara T., Sakamoto A., Kishi C., Waguri S., Eishi Y., Hino O., Tanaka K., Mizushima N. (2011). Autophagy-deficient mice develop multiple liver tumors. Genes Dev.

